# Novel Multi-Classification Dynamic Detection Model for Android Malware Based on Improved Zebra Optimization Algorithm and LightGBM

**DOI:** 10.3390/s24185975

**Published:** 2024-09-14

**Authors:** Shuncheng Zhou, Honghui Li, Xueliang Fu, Daoqi Han, Xin He

**Affiliations:** College of Computer and Information Engineering, Inner Mongolia Agricultural University, Hohhot 010018, China; zhousc1121@163.com (S.Z.); fuxl@imau.edu.cn (X.F.); 15623587331@163.com (D.H.); hx_121382024@163.com (X.H.)

**Keywords:** Android malware detection, improved zebra optimization algorithm, LightGBM, hyperparameter optimization

## Abstract

With the increasing popularity of Android smartphones, malware targeting the Android platform is showing explosive growth. Currently, mainstream detection methods use static analysis methods to extract features of the software and apply machine learning algorithms for detection. However, static analysis methods can be less effective when faced with Android malware that employs sophisticated obfuscation techniques such as altering code structure. In order to effectively detect Android malware and improve the detection accuracy, this paper proposes a dynamic detection model for Android malware based on the combination of an Improved Zebra Optimization Algorithm (IZOA) and Light Gradient Boosting Machine (LightGBM) model, called IZOA-LightGBM. By introducing elite opposition-based learning and firefly perturbation strategies, IZOA enhances the convergence speed and search capability of the traditional zebra optimization algorithm. Then, the IZOA is employed to optimize the LightGBM model hyperparameters for the dynamic detection of Android malware multi-classification. The results from experiments indicate that the overall accuracy of the proposed IZOA-LightGBM model on the CICMalDroid-2020, CCCS-CIC-AndMal-2020, and CIC-AAGM-2017 datasets is 99.75%, 98.86%, and 97.95%, respectively, which are higher than the other comparative models.

## 1. Introduction

In the intelligent era, the rapid development and diversification of Android applications have led to an increase in Android malware attacks, which have become increasingly severe [1]. Traditional Android malware detection methods based on static analysis, especially signature-based techniques, can effectively identify known Android malware. However, when the code structure and behavioral patterns of Android malware change, static analysis-based detection methods find it difficult to effectively identify their true intent [2]. This leads to a decrease in detection accuracy.

For this reason, researchers have proposed a dynamic analysis method that focuses on the behavior of Android malware [3]. Android malware is detected through the results of its execution in a sandbox environment. To improve the accuracy of Android malware detection, machine learning (ML) algorithms have been widely adopted. Aslan et al. [2] demonstrated that ML methods lead to improved detection of both known and unknown Android malware. Recently, Gradient Boosting Decision Trees (GBDTs) [4] have shown excellent performance in many studies [5]. Among these methods is the Light Gradient Boosting Machine (LightGBM) [6], an improved GBDT with the advantages of faster training and higher efficiency [7]. In a study by Kirubavathi et al. [7], LightGBM achieved an accuracy of 98.05% in Android malware detection.

However, the LightGBM model contains a large number of hyperparameters, and the values of these hyperparameters significantly affect the model’s detection performance. Manual parameter tuning is extremely challenging due to the sheer number of hyperparameters, the model’s inherent complexity, and the interdependencies among these non-linear hyperparameters. In order to construct an efficient LightGBM-based Android malware detection model, this paper proposes using an optimization method to set the values of the LightGBM hyperparameters.

Since hyperparameter optimization problems are usually nonconvex or nondifferentiable optimization problems, traditional optimization methods may obtain local rather than global optimal solutions. In contrast, metaheuristic algorithms can be effective techniques for solving complex, large-search-space, and nonconvex optimization problems belonging to hyperparametric optimization problems [8]. The Zebra Optimization Algorithm (ZOA) is a novel metaheuristic algorithm that has demonstrated better performance compared to traditional metaheuristics [9]. Despite this, the ZOA still suffers from issues of slow convergence and the tendency to fall into local optimal solutions, limiting its performance.

This paper introduces a dynamic analysis detection model for Android malware, utilizing the Improved Zebra Optimization Algorithm (IZOA) and LightGBM, named the IZOA-LightGBM model, aimed at effectively enhancing the accuracy of malware detection. The model improves the initialization process of the ZOA by introducing elite opposition-based learning (EOBL), which expands the search space and accelerates the convergence speed of the algorithm. The population updating process of the ZOA is improved by introducing the firefly disturbance strategy (FDS), which enhances the local search ability and helps the algorithm avoid falling into local optimal solutions. The optimization process of the hyperparameters of the LightGBM model is completed using the IZOA, which ultimately enables the dynamic detection of Android malware. The primary contributions of this study are outlined in the following points:

1. A novel dynamic analysis-based Android malware detection model using the improved ZOA and LightGBM is proposed, called the IZOA-LightGBM model. It effectively improves the accuracy of Android malware detection.
2. The EOBL and FDS are introduced to improve the traditional ZOA and enhance its search capabilities. The improved algorithms are used to optimize the hyperparameters of the LightGBM model.
3. A suite of experiments was designed to explain and validate the proposed model.

The rest part of this paper is organized as follows. Section 2 presents related work in the field of Android malware detection. Section 3 presents the IZOA-LightGBM model proposed in this paper. Section 4 evaluates and analyzes the detection model proposed in this paper. Section 5 summarizes this paper and provides potential directions for future research.

## 2. Related Work

Android malware detection methods can generally be divided into three categories: static analysis methods, dynamic analysis methods, and hybrid analysis methods. The results of a study by Gorment et al. [10] in 2023 showed that most of the studies used static analyses with 53.3%, followed by dynamic analyses with 28.9%, and hybrid analyses with only 17.8%.

Static analysis methods, also known as code analysis methods, involve examining software code while it is in a non-executing state. This approach gathers static data from the code to determine if the software in question is classified as Android malware. Researchers have proposed various static analysis-based methods for Android malware detection. For instance, Khariwal et al. [11] used information gain to rank permissions and intents with the aim of finding the optimal set of permissions and intents that would enable better detection of Android malware and proposed a new algorithm by applying several ML algorithms such as Support Vector Machine (SVM), Random Forests (RF), Naive Bayes (NB), and K-Nearest Neighbors (KNN) to find the optimal set. The experiments displayed that RF achieved the highest accuracy rate of 94.73%. Dhalaria et al. [12] extracted API call attributes from the classes.dex file, as well as permission and intent attributes from the AndroidManifest.xml file, and then used these attributes for training and testing to classify the application. The results of the study showed that combined features perform better compared to individual features and it was found that the RF and KNN classifiers provided the highest accuracy of 95.9% for classifying Android apps. Shatnawi et al. [13] proposed an Android malware detection method based on Android permissions and API calls. The experimental results showed that the SVM classifier achieved the highest recognition rate among other comparative classifiers. An average accuracy of 94% was achieved with permission features, while 83% was achieved with API call features. However, when complex obfuscation techniques (e.g., code obfuscation, shelling) are used on an application, static analysis might fail to uncover the genuine behavior of the malware.

Dynamic analysis methods, also known as behavioral analysis methods, record behavioral information such as behavioral logs, context parameters, API call sequences, and other behavioral information of Android malware by executing Android malware samples in a virtual controlled environment. Li et al. [14] proposed a novel framework for Android malware detection based on deep learning (DL) and dynamic analysis. The framework first applies embedding and convolutional layers to perform a joint representation of multiple APIs to represent software behavior. Second, the semantic information of each API call is represented using the API’s category, activity, and operation objects. Finally, a bi-directional long and short-term memory network module is used to mine the relational information between APIs. The study’s findings demonstrated that the method achieves an accuracy rate of 97.31%. Chai et al. [15] proposed a dynamic prototype network for small-sample Android malware detection. This network first uses a dynamic convolutional network to extract dynamic features based on sample adaptation. Subsequently, a dual-sample dynamic activation function is introduced to leverage the inter-sample correlation, thereby mitigating the influence of features that lack correlation between samples on the metric learning process. Experiments showed that the accuracy of the method reaches 94.32%. Hwang et al. [16] proposed a two-stage ransomware detection method based on dynamic analysis. The method first uses a Markov model to capture the features of the software, and later uses an RF model for detection. Experiments showed that the accuracy of the method is 97.3%. Mahdavifar et al. [17] proposed a dynamic Android malware detection method using semi-supervised DL. This method is trained using a set of labeled and a set of unlabeled samples and uses dynamic analysis to extract dynamic behavioral profiles as feature vectors. Experimental results showed that the method achieves up to 96.7% accuracy. However, dynamic analysis methods require more computational resources and time because they require malware execution in an isolated environment.

Hybrid analysis methods integrate static analysis and dynamic analysis methods to capitalize on the strengths of each approach. Static analysis methods are first used to examine the Android malware. Then, the Android malware is launched in a simulated environment to observe its actual behavior. Hadiprakoso et al. [18] trained various ML algorithms including SVM, Decision Tree (DT), RF, NB, and KNN to observe their performance on datasets based on both static and dynamic analysis. Experimental results showed that hybrid analysis can improve Android malware detection accuracy by 5% compared to static analysis alone. Ding et al. [19] proposed a model based on hybrid analysis, which first uses a chi-square test for feature selection, followed by RF for classification, and then uses a residual network combined with a long short-term memory network on top of RF classification results for further classification. Experiments showed that the model has an accuracy of 99%. Amer et al. [20] introduced a universal behavioral graph framework to characterize both malicious and benign processes. The behavioral graph model is based on a combination of statistical, contextual, and graph mining attributes that detect both explicit and implicit connections among API functions within a sequence of invocations. Experiments showed that the model achieves 97.7% accuracy. However, the hybrid analysis method might demand additional computational resources and time because it merges static and dynamic analysis and needs to harmonize and integrate the results of the two analysis methods.

In addition, some scholars have suggested Android malware detection methods that utilize improved meta-heuristic algorithms. For instance, Al-Ogaili et al. [21] suggested to improve the population update process of the whale optimization algorithm using weighted arithmetic mean and applied the improved algorithm for feature selection to detect Android malware attacks. Dong et al. [22] proposed a method to improve the initialization process of the firework optimization algorithm using Lévy flights and improved the updating process of the algorithm using an elitist bootstrapping strategy. The improved fireworks optimization algorithm was then applied to optimize the hyperparameters of the SVM model, subsequently, the optimized SVM model was used to detect Android malware. Aldehim et al. [23] proposed an initialization process to improve the black widow optimization algorithm using Gaussian chaotic mapping for feature selection. Liu et al. [24] introduced a probability density function to improve the particle update process of the particle swarm optimization (PSO) [25] algorithm and applied the improved algorithm to prevent malicious attacks. Given that the ZOA is a relatively new optimization algorithm, fewer researchers have contributed improvements. For example, Qi et al. [26] used chaotic mapping to improve the ZOA population initialization process and improved the population updating process of the algorithm using a positive cosine strategy and dynamic adaptive weighting coefficients. DAMA et al. [27] used chaotic sinusoidal mapping to improve the population updating process of the ZOA. Nevertheless, these approaches continue to face issues with slow convergence rates and a propensity for becoming stuck in local optima.

## 3. Methods

**Figure 1:**
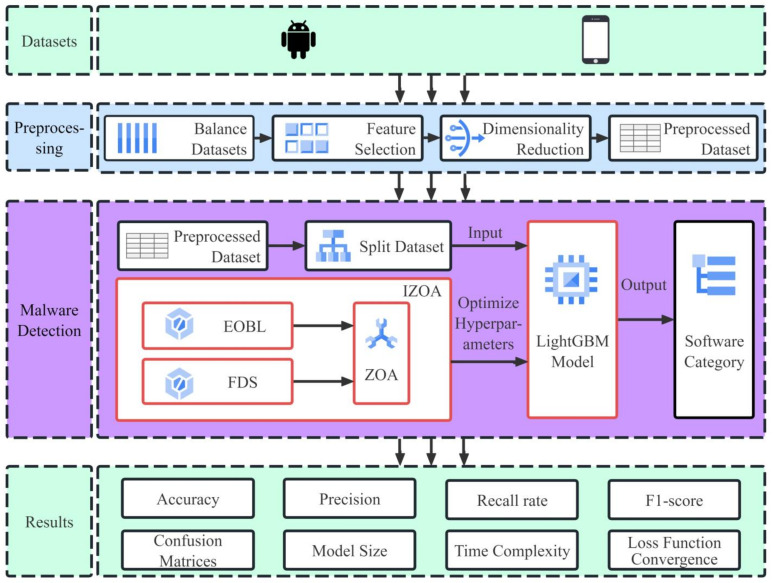
Structural framework of IZOA-LightGBM model.

LightGBM, proposed by Ke and colleagues in 2017, is an improvement of the GBDT algorithm [6]. It diminishes computational demands by employing gradient-based one-side sampling, which filters out samples with smaller gradients during training. In addition, the use of the exclusive feature bundling algorithm reduces the number of features used in the model training process and effectively reduces the complexity of the model. The model has received a lot of attention and has been successfully used for many different types of tasks such as classification, regression and ranking [28]. The LightGBM model has the advantages of faster training and higher efficiency [7]; these advantages make it ideal for Android malware detection. However, the performance of LightGBM is heavily influenced by its many hyperparameters. In order to better utilize LightGBM for Android malware detection, this study used the IZOA to optimize the hyperparameters of the LightGBM model. Figure 1 illustrates the structural framework of the method proposed in this paper. Table 1 lists 11 key hyperparameters that require optimization in the LightGBM model [28,29].

The content of this chapter is arranged as follows: Section 3.1 introduces the hyperparameter optimization method for the LightGBM model based on the ZOA; Section 3.2 introduces the process of optimizing hyperparameters with the improved ZOA, namely IZOA; and Section 3.3 offers an in-depth explanation of the IZOA-LightGBM model for the dynamic analysis and detection of Android malware.

### 3.1. Zebra Optimization Algorithm

The ZOA, introduced by Trojovska et al. [9] in 2022, is a meta-heuristic optimization algorithm. The algorithm solves the optimization problem by simulating the foraging and defensive behaviors of zebras. The ZOA outperforms nine well-known optimization algorithms, such as the Grey Wolf Optimizer (GWO) [30], Genetic Algorithm (GA) [31], PSO [9], etc. The hyperparameter steps for optimizing LightGBM based on the conventional ZOA are as follows:

1. Initialize zebra population: Randomly generate an initial population containing z zebras. Each zebra i is defined as a position vector $A_{i}=(a_{i,1},{\cdots ,a}_{i,j},\cdots ,a_{i,11})$ that corresponds to the 11 hyperparameter values to be optimized in the LightGBM model as described above. $a_{i,j}$ is the jth hyperparameter value, which takes the value interval $\left[l_{j},u_{j}\right]$, 1≤i≤z, 1≤j≤11. The fitness function $F\left(A_{i}\right)$ is specified as depicted in Equation (1):
  (1) $$F\left(A_{i}\right)=\min\left(1-{Accuracy}_{LightGBM}\right)$$
  where ${Accuracy}_{LightGBM}$ denotes the accuracy of the LightGBM model.

The zebra with the lowest fitness function value in the population is defined as the Pioneer Zebra (PZ). The PZ guides the other zebras toward its position during the optimization process.

2. Foraging phase: In this phase, the jth hyperparameter value of zebra $A_{i}$ is calculated using Equation (2). Subsequently, the position is updated using Equation (3):
  (2) $$a_{i,j}^{new}=a_{i,j}+r\left({PZ}_{j}-\varphi \cdot a_{i,j}\right)$$
  (3) $$A_{i}=\left\{\begin{array}{l} A_{i}^{new},\;\;\;\;F\left(A_{i}^{new}\right)<F\left(A_{i}\right)\\ A_{i}\;\;\;\;,\;\;\;\;else \end{array}\right.$$
  where $A_{i}^{new}$ is the new position of zebra $A_{i}$, $a_{i,j}^{new}$ is the updated value of its jth hyperparameter value, and $F\left(A_{i}^{new}\right)$ is the updated value of the corresponding fitness function. r represents a random value within the range [0, 1], ${PZ}_{j}$ is the jth hyperparameter value of the pioneer zebra, and φ=round(1+r).
3. Defense phase: In this phase, the zebra either flees upon being attacked by a lion (M1) or it counterattacks (M2). Let the selection probability, p∈[0, 1], be a random number that determines the strategy. The zebra’s position is then updated based on the chosen strategy, as described by Equation (4). Subsequently, update the zebra position using Equation (3).
  (4) $$a_{i,j}^{new}=\left\{\begin{array}{l} M1:a_{i,j}+\beta \cdot (2r-1)\cdot (1-\frac{t}{T})\cdot a_{i,j},\;\;\;\;p\leq 0.5\\ M2:\;a_{i,j}+r\cdot \left({AZ}_{j}-\varphi \cdot a_{i,j}\right)\;\;\;\;\;\;\;\;\;\;\;\;\;\;\;\;,\;\;\;\;else \end{array}\right.$$
  where t is the current iteration number, T is the maximum iteration number, and β is a constant value equal to 0.01 [9]. AZ denotes the position of any zebra in the zebra population except the ith one, and ${AZ}_{j}$ is the value of its jth hyperparameter.
4. Iteration: In each iteration, the zebra with the lowest fitness value is chosen as the pioneer zebra PZ. Steps 2 and 3 are reiterated until the iteration limit is attained. The PZ selected after the last iteration is considered the optimal solution.

### 3.2. Improved Zebra Optimization Algorithm

In the ZOA, the initialization population is generated randomly, which can lead to slow convergence [26]. This study improves the initialization process of the ZOA by introducing EOBL, which accelerates the convergence speed and boosts the algorithm’s global search capability. In addition, the ZOA may also fall into local optimal solutions during the population updating process [27]. In this paper, the FDS is introduced to improve the population updating process of the ZOA, further avoiding falling into local optimal solutions and enhancing the local search capability.

#### 3.2.1. Introducing Elite Opposition-Based Learning to Improve Population Initialization

Opposition-based learning (OBL) [32] is a strategy for enhancing ML proposed by Tizhoosh et al. in 2005. Wang et al. introduced the idea of elite learning and proposed EOBL based on OBL [33]. Experimental results demonstrated that the EOBL has better performance than OBL [33].

In this study, elite opposition-based zebras were generated through EOBL. The fitness function values of both the current and elite opposition-based zebras were calculated and compared, and the zebra with the lower value was selected for the subsequent generation. The detailed steps are outlined below:

1. For any zebra $A_{i}$, its opposition-based zebra is denoted by $A_{i}^{\prime }=\left(a_{i,1}^{\prime },\cdots ,a_{i,j}^{\prime },\cdots ,a_{i,11}^{\prime }\right)$, where $a_{i,j}^{\prime }={r\cdot (l}_{j}+u_{j})-a_{i,j}$. If $F(A_{i}^{\prime })<F(A_{i})$, then $A_{i}$ is defined as an elite zebra, denoted as $E_{i}$. Otherwise, $A_{i}$ is directly used as a next-generation zebra.
2. All elite zebras in the zebra population are selected to form the elite zebra population. Let $e_{i,j}$ be the jth hyperparameter value of the elite zebra $E_{i}$; then, the jth hyperparameter value $e_{i,j}^{*}$ of the elite opposition-based zebra $E_{i}^{*}$ is calculated as shown in Equation (5). Use Equation (6) to select the next generation of zebras.
  (5) $$e_{i,j}^{*}=r\cdot \left(a_{j}+b_{j}\right)-e_{i,j}$$
  (6) $$A_{i}=\left\{\begin{array}{l} E_{i}^{*}\;,\;\;\;\;F\left(E_{i}^{*}\right)<F\left(A_{i}\right)\\ A_{i}\;,\;\;\;\;else \end{array}\right.$$
  where $a_{j}$ and $b_{j}$ are the lower and upper bounds of the elite zebra population, respectively. $a_{j}=min[e_{1,j},\cdots ,e_{i,j},\cdots ,e_{p,j}]$*,* $b_{j}=max[e_{1,j},\cdots ,e_{i,j},\cdots ,e_{p,j}]$, and p denotes the number of elite zebras, which is less than or equal to the total zebra count within the population. $F\left(E_{i}^{*}\right)$ is the value of the fitness function corresponding to the elite opposition-based zebra $E_{i}^{*}$.

**Figure 2:**
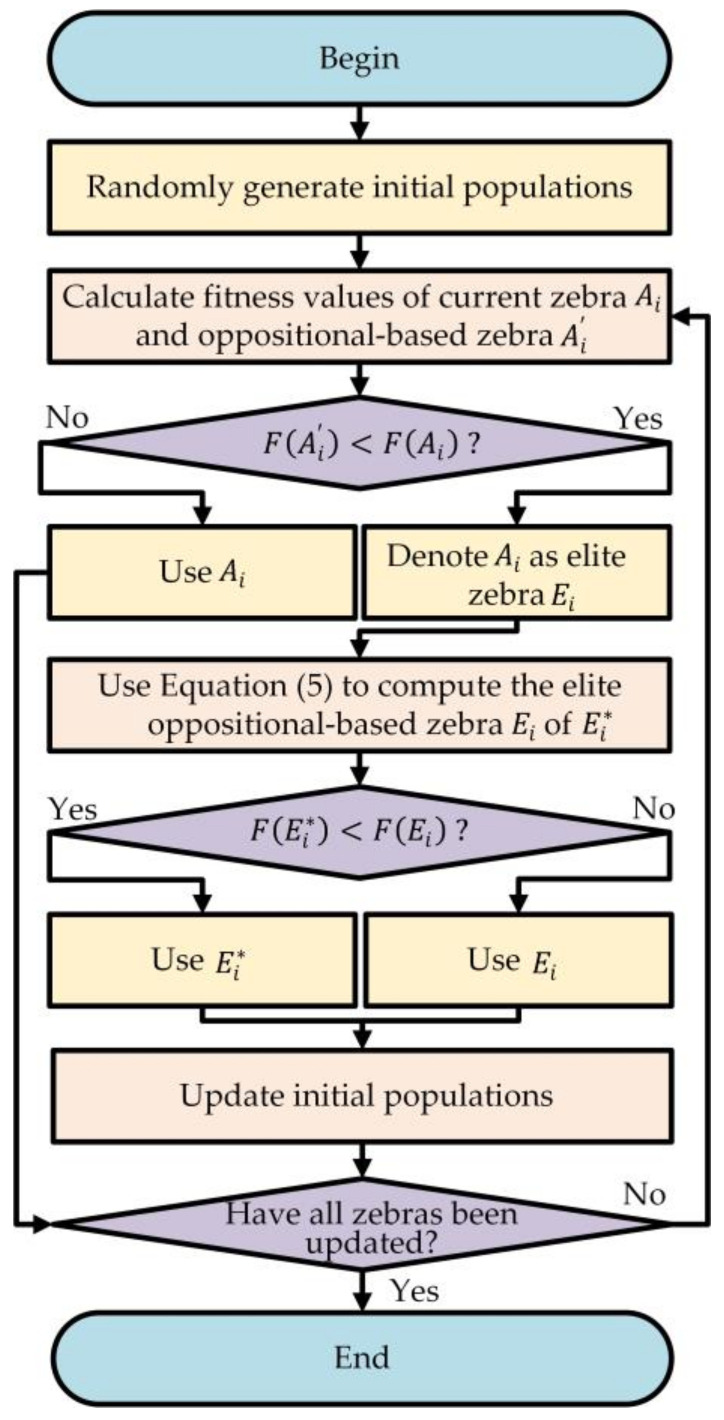
Flowchart of the introduction of EOBL for improved population initialization.

Figure 2 shows the population initialization process based on the EOBL improvement of the ZOA for optimization of the LightGBM hyperparameters.

#### 3.2.2. Introducing Firefly Disturbance Strategy to Update Zebra Positions

The firefly algorithm, introduced by Yang et al. [34] in 2010, is a meta-heuristic algorithm. The idea originates from the attraction and movement between individual fireflies in nature. This paper introduces the FDS, a key strategy in the firefly algorithm, to improve the population update process of the ZOA. Through the FDS, new zebras are generated, the fitness function values of the current zebra and the new zebra are compared, and the lower one is selected as the next-generation zebra.

Following the position adjustment in the defense phase, the position of zebra $A_{i}$ is updated again using the FDS. The jth hyperparameter value of zebra $A_{i}$ is calculated using Equation (7), and the position is updated using Equation (3):

(7) $$a_{i,j}^{new}=a_{i,j}+\beta \cdot \left({PZ}_{j}-a_{i,j}\right)+\delta \cdot \left(r-\frac{1}{2}\right)$$

where $\beta =\beta_{0}\cdot e^{-\theta {S_{A_{i},PZ}}^{2}}$, $\beta_{0}$, and θ are constants; $S_{A_{i},PZ}$ is the spatial distance between the current zebra $A_{i}$ and the pioneer zebra PZ; and δ is the step factor belonging to the interval $\left[0,\;1\right]$.

**Figure 3:**
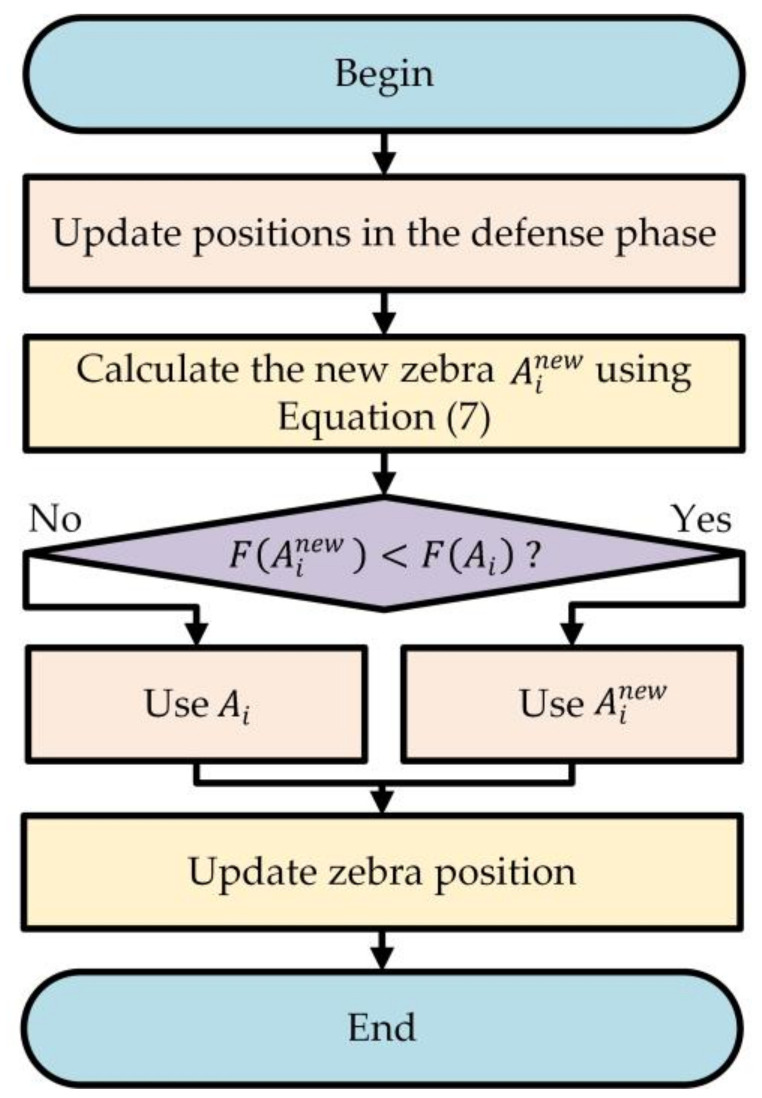
Flowchart of the introduction of FDS to improve zebra position updating.

Figure 3 illustrates the process of population updating based on the FDS to improve the ZOA.

#### 3.2.3. Detailed Steps to Improve the Zebra Optimization Algorithm

**Figure 4:**
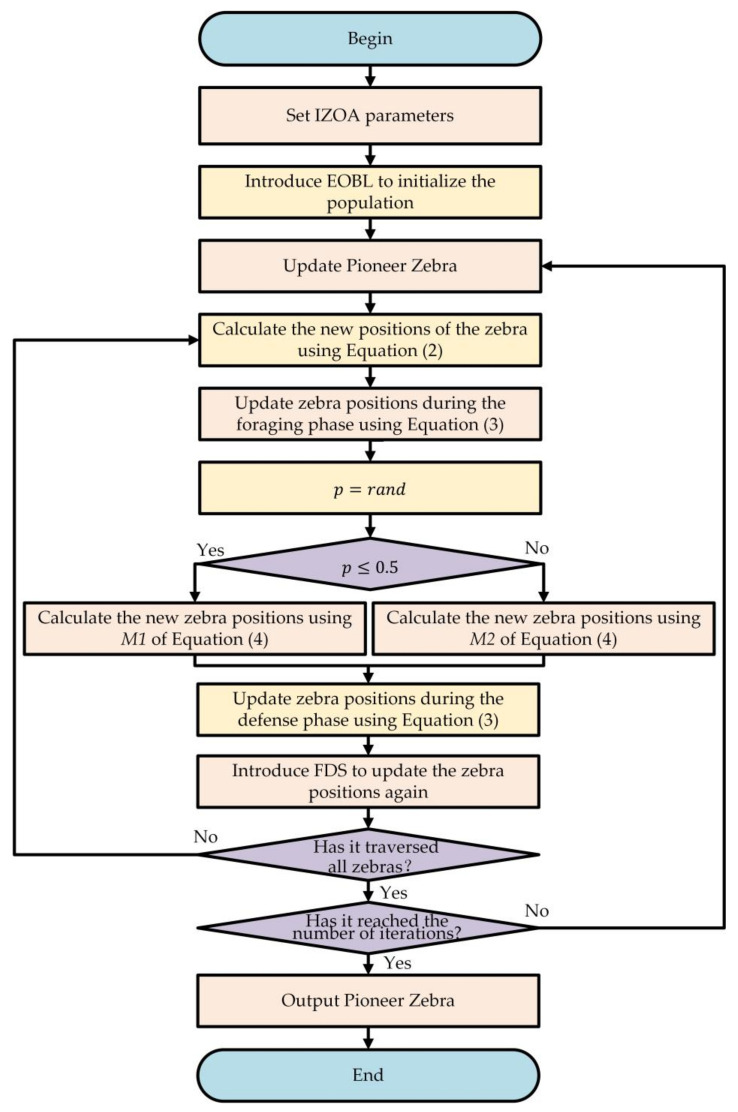
Flowchart of the IZOA.

This section describes in detail the complete steps of the IZOA for optimizing the hyperparameters of LightGBM. The flowchart of the IZOA is shown in Figure 4.

The following section describes the specific steps of the IZOA:

1. Setting IZOA parameters: Set the parameters of the IZOA, including the number of zebra population, the minimum and maximum values of hyperparameters to be optimized in the LightGBM model, the maximum number of iterations, and so on.
2. Introducing EOBL to initialize the population: First, randomly generate the zebra population. Then, compare the fitness function values of current zebras and elite opposition-based zebras, selecting the one with the lower value as the next-generation zebras.
3. Update the pioneer zebra: Select the zebra with the lowest fitness function value in the population as the pioneer zebra.
4. Position update during foraging phase: Calculate the new position of the zebra using Equation (2) and update the position using Equation (3). If the new position has a lower fitness function value, update the position; otherwise, retain the original position.
5. Defense phase position update: Calculate the new position of the zebra using Equation (4) based on the value of the random number p. Update the position using Equation (3). If the new position has a lower fitness function value, update the position; otherwise, retain the original position.
6. Introduces the FDS to update the zebra position again: Calculate the new position of the zebra using Equation (7) and update the position using Equation (3). If the new position has a lower fitness function value, update the position; otherwise, retain the original position.
7. Check whether all zebras are traversed: If the current iteration has not traversed all zebras, return to step 4 to update the next zebra’s position. If all zebras have been updated, proceed to the next step.
8. Verify if the iteration limit has been met: If the maximum number of iterations has not been reached, return to step 3 to continue iterating. If reached, output the pioneer zebra of the IZOA, i.e., the optimal hyperparameters of the LightGBM model, and end the optimization process.

### 3.3. Android Malware Detection Model IZOA-LightGBM

**Figure 5:**
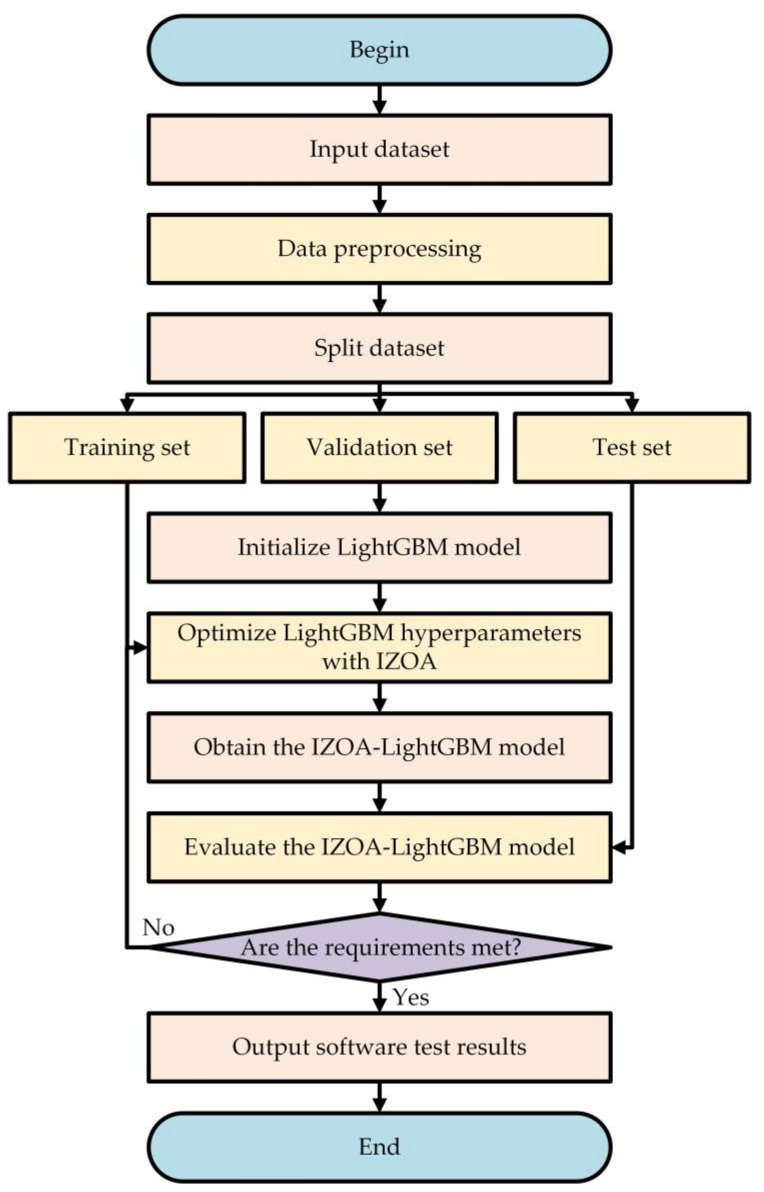
Flowchart of the IZOA-LightGBM model.

The IZOA-LightGBM model utilizes the IZOA to tune the hyperparameters of LightGBM with the aim of identifying the optimal hyperparameter settings to improve the detection accuracy. Figure 5 illustrates the flowchart of the IZOA-LightGBM model.

The specific steps for constructing the IZOA-LightGBM model are as follows:

1. Begin: Initiate the optimization process and initialize the environment and parameters.
2. Input dataset: Input Android malware dataset based on dynamic analysis.
3. Data preprocessing: Convert the dataset into a format acceptable to the model and perform data preprocessing to enhance model performance.
4. Split the dataset: Divide the dataset into training, validation, and test sets for respective uses in model training, parameter tuning, and performance evaluation.
5. Initialize LightGBM model: Initialize the LightGBM model with default hyperparameters.
6. Optimize the LightGBM model hyperparameters with the IZOA: Use the IZOA to optimize the LightGBM model hyperparameters, evaluate different combinations using the validation set, and pass the selected optimal hyperparameters to the LightGBM model.
7. Obtain the IZOA-LightGBM model: Obtain the IZOA-LightGBM model with optimal hyperparameters.
8. Evaluate the IZOA-LightGBM model: Assess the IZOA-LightGBM model performance with the test set. Calculate the various metrics of the model (e.g., accuracy, recall, F1-score, etc.). If the model meets expectations, proceed to the next step; otherwise, return to step 6 and re-optimize.
9. Output software test results: Output the final model test results and indicators.
10. End: End the testing process, save all necessary results and models.

The pseudo-code of the IZOA-LightGBM model is shown in Algorithm 1.
**Algorithm 1:** Pseudo-Code of IZOA-LightGBMStart the IZOA-LightGBM model.Input: Dataset.Perform data preprocessing.Initialize the LightGBM model.Define the IZOA fitness function using Equation (1).Set the number of iterations (T), the number of zebras’ population (z), and the dimension of the problem to be optimized for the IZOA.Use EOBL, i.e., Equations (6) and (7), to create the initial zebra population.Evaluate the fitness values of the zebras.for k=1 : T  Update the Pioneer Zebra (PZ)  for i=1 : z   #Phase 1   Calculate the new position of the ith zebra using Equation (2).   Update the position of the ith zebra using Equation (3).   #Phase 2   p=rand()   if p≤0.5:    Calculate the new position of the ith zebra using $M_{1}$ of Equation (4).   else:    Calculate the new position of the ith zebra using $M_{2}$ of Equation (4).   end if   Update the position of the ith zebra using Equation (5).   Use the FDS, i.e., Equations (8) and (9), to update the position of the ith zebra again.   Save the PZ.  end for i=1 : zend for k=1 : TInput the PZ obtained by the IZOA into the LightGBM model.Obtain the LightGBM model with optimal hyperparameters.Evaluate the IZOA-LightGBM model.Use the optimal IZOA-LightGBM model for detection.End the IZOA-LightGBM model.

## 4. Experiment and Analysis

Through extensive experiments, this study evaluated the performance of the proposed IZOA-LightGBM model. The system’s hardware was equipped with an Intel (R) Core (TM) i7-6700 CPU at 3.40 GHz, with 16.0 GB of RAM. The software setup incorporated Windows 10, Visual Studio Code, and Python 3.11.2. In this section, Section 4.1 describes the dataset used for the evaluation. Section 4.2 details the evaluation metrics. Section 4.3 outlines the preprocessing steps for the dataset. Section 4.4 presents and analyzes the evaluation results of the IZOA-LightGBM model in detail. Section 4.5 summarizes these results.

### 4.1. Datasets

Three widely recognized public datasets based on dynamic analysis are used to validate the performance of the IZOA-LightGBM model proposed in this paper on different datasets. The first is the Canadian Institute for Cybersecurity Android Malware 2020 (CICMalDroid-2020) dataset [17,35], abbreviated as the CMD dataset for ease of writing. The second is the Canadian Institute for Cybersecurity project in collaboration with the Canadian Centre for Cyber Security Android Malware 2020 (CCCS-CIC-AndMal-2020) dataset [36,37], referred to as the CCA dataset for ease of writing. The third is the Canadian Institute for Cybersecurity Android Adware and General Malware (CIC-AAGM-2017) dataset [38], referred to as the AAGM dataset for ease of writing. These two datasets are ideal for Android malware detection evaluation due to their high quality and diversity [39].

#### 4.1.1. CMD Dataset

The CMD dataset comprises over 17,341 Android samples from various sources, such as AMD, MalDozer, VirusTotal, and the Contagio security blog. For dynamic analysis, the dataset covers five different categories: adware, banking malware, SMS malware, riskware, and benign software. This dataset contains 471 features. Table 2 displays the sample counts for each category.

#### 4.1.2. CCA Dataset

The CCA dataset, which is a publicly available dataset jointly produced by the Canadian Center for Cybersecurity and the Canadian Cybersecurity Institute in 2020. For dynamic analysis, the dataset contains 142 features. There are 14 different types of Android malware categories in this dataset, including adware, backdoor, file infector, no category, Potentially Unwanted Application (PUA), ransomware, riskware, scareware, Trojan, Trojan banker, Trojan dropper, Trojan SMS, Trojan spy, and zero-day vulnerability. However, the authors of the dataset recommend excluding the no category and zero-day vulnerability categories because the data for these categories are incomplete. After removing the no category and zero-day vulnerability categories from the dataset, 12 categories remained. Table 3 displays the sample counts for each category.

#### 4.1.3. AAGM Dataset

The AAGM dataset was compiled by the Canadian Institute for Cyber Security Research. To generate traffic that is representative of the real world, the institute used an Android smartphone (Nexus 5) rather than an emulator or Android virtual device for traffic collection, ensuring both the quality and quantity of the dataset. For dynamic analysis, the dataset contains 80 features. There are three different types of Android malware categories in this dataset: 1500 benign, 250 adware, and 150 general malware. The number of traffic bars for each category is shown in Table 4.

### 4.2. Evaluation Metrics

The following metrics were utilized to assess the model performance:

1. Training time [17]: The training time is the time taken by the model from the beginning to the end of the training process.
2. Detection time [17]: The detection time is the time taken by the model from receiving the sample to be detected to making a judgment on whether it is malware or not.
3. Model size [17]: The model size is the amount of space occupied by model on the storage medium.
4. Accuracy [40]: Accuracy is the ratio of the correctly identified samples to the total number of samples in the model. The formula is presented in Equation (8):
  (8) $$Accuracy=\frac{TP+TN}{TP+FP+TN+FN}$$
  where TP (True Positive) represents the number of samples that the model correctly predicted as a category; TN (True Negative) represents the number of samples that the model correctly predicted as other categories; FP (False Positive) represents the number of samples that the model incorrectly predicted as samples from other categories; and FN (False Negative) represents the number of samples that the model incorrectly predicts as other categories for that category.
5. Precision [40]: Precision is the proportion of samples that the model classified as positive that are actually of the positive category. The formula is presented in Equation (9):
  (9) $$Precision=\frac{TP}{TP+FP}$$
6. Recall rate [40]: The recall rate is the ratio of the number of samples correctly identified as positive by the model to the total number of samples that are actually positive. The formula is presented in Equation (10):
  (10) $$Recallrate=\frac{TP}{TP+FN}$$
7. F1-Score [40]: The F1-score is a reconciled average of the precision and recall and is used to measure the comprehensive performance of the model in the classification task. The formula is presented in Equation (11):
  (11) $$F1-Score=2\times \frac{Precision\times Recall}{Precision+Recall}$$

### 4.3. Data Preprocessing

This study conducted a series of data preprocessing steps on the CMD and CCA datasets, including dataset balancing, feature selection, and dimensionality reduction. Balancing the dataset made the model predictions fair for all categories, while making the model generalize better for all categories [41]. The feature selection and feature dimensionality reduction were designed to remove irrelevant or redundant features. In doing so, the model complexity is diminished. In most cases, this approach improves the detection accuracy of the model while also reducing the risk of model overfitting [42].

#### 4.3.1. Balancing Datasets

This study employed three oversampling techniques to balance the datasets: the Adaptive Synthetic Sampling Approach (ADASYN) [43], Random Oversampling Approach (ROA) [41], and Synthetic Minority Over-sampling Technique (SMOTE) [44]. For the CMD dataset, the SMS malware category has the largest number of samples (3904), so this study oversampled each category to 4000 samples. For the CCA dataset, the Trojan category has a larger number of samples, totaling 4025. This study oversampled the categories with fewer than 4025 samples to 4025 samples, and undersampled the categories with more than 4025 samples using random undersampling to 4025 samples. The AAGM dataset was balanced to 10,000 entries per category due to the large number of samples and the limited computing power of the experimental equipment. Table 5 shows the accuracy of the datasets generated using different oversampling techniques on the default LightGBM model.

As can be seen from Table 5, for both the CMD, CCA, and AAGM datasets, the oversampling technique generally improves the accuracy of the models, compared to the unbalanced dataset. Both CMD and CCA datasets achieved the highest accuracy using the ROA. The AAGM dataset achieved the highest accuracy using the Adasyn oversampling technique.

#### 4.3.2. Feature Selection

In this study, the balanced CMD, CCA, and AAGM datasets were fed into the LightGBM model with default hyperparameters. The purpose was to determine the importance of features for predicting labels through feature selection. In this study, split gain [6] was used as a measure of feature importance. Using this method, feature columns that have low split gain are identified and retained, and they are combined with label columns to construct a new dataset. The results before and after feature selection are shown in Table 6.

As shown in Table 6, the split gain-based feature selection strategy decreases the feature count of the datasets and marginally improves the model accuracy. This may be due to the fact that the strategy effectively removes redundant features that do not contribute much to the detection results, as well as reduces the noise interference in the data, thus allowing the model to focus more on the information that contributes substantially to the detection results. 

#### 4.3.3. Feature Dimensionality Reduction

**Figure 6:**
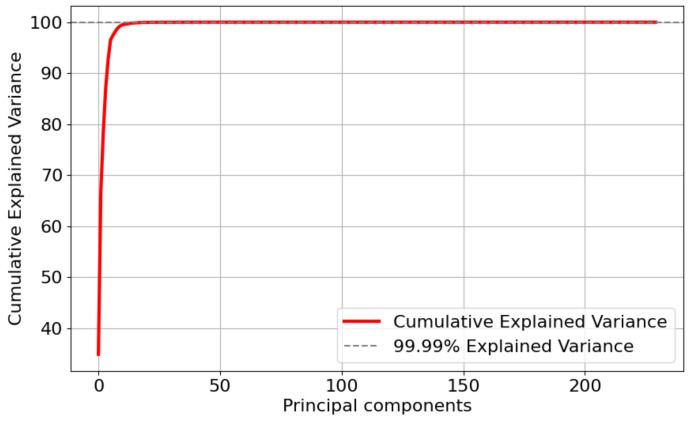
Variance distribution of the number of principal components of the CMD dataset.

**Figure 7:**
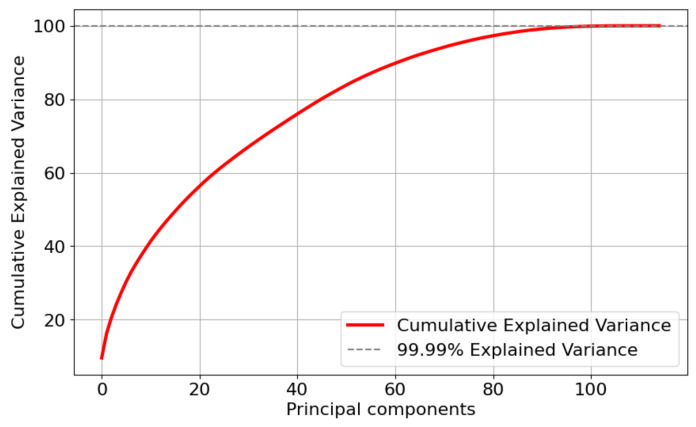
Variance distribution of the number of principal components of the CCA dataset.

**Figure 8:**
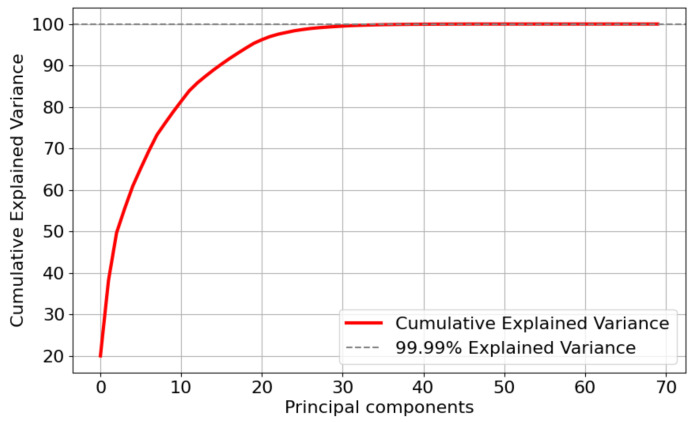
Variance distribution of the number of principal components of the AAGM dataset.

This study further applied Principal Component Analysis (PCA) [42] to the after feature selection dataset for feature dimensionality reduction. PCA is a method of dimensionality reduction that transforms the high-dimensional space into a low-dimensional one, aiming to preserve the original data structure and important information. Reducing the data dimensions can enable ML models to analyze data more quickly and efficiently. Figure 6, Figure 7 and Figure 8 show the cumulative explained variance for the CMD, CCA, and AAGM datasets, respectively. The cumulative explained variance refers to the proportion of the total variance explained by all principal components that is accounted for by the first n principal components. The results before and after dimensionality reduction are shown in Table 7.

Figure 6 and Table 7 indicate that 99.9% of the cumulative explained variance was achieved with 29 principal components after conducting dimensionality reduction on the CMD dataset. Although the accuracy was reduced by 0.7%, the training time was reduced by 1.5 s. In addition, Figure 7 and Table 7 indicate that 99.9% of the cumulative explained variance was achieved with 108 principal components after dimensionality reduction processing on the CCA dataset. Although the accuracy was reduced by 0.52%, the training time was reduced by 1.2 s. Figure 8 and Table 7 indicate that 99.9% of the cumulative explained variance was achieved with 108 principal components after dimensionality reduction processing on the AAGM dataset. Although the accuracy was reduced by 1.2%, the training time was reduced by 1.5 s.

### 4.4. Experimental Results and Analysis

To ensure the reliability of the experimental results and to exclude the influence of chance factors, all experiments in this section were repeated 20 times independently in study paper, and the final results were taken as the average of these independent experiments.

When learning dependencies from data, it is crucial to categorize the data into training, validation, and test sets to prevent overfitting [20]. In this study, 80% of the dataset was utilized for model training, 10% was used for validation to adjust the model, and the remaining 10% was allocated for testing and evaluating the model. To further prevent model overfitting, an early stop strategy was used throughout the evaluation process, establishing 500 as the training epoch count, and ending the training when the model accuracy did not improve for 10 consecutive rounds.

During the experiments, some classical optimization algorithms and classifiers were selected as comparison models, including the LightGBM model optimized using the ZOA (ZOA-LightGBM), the LightGBM model optimized using the Simulated Annealing (SA) algorithm (SA-LightGBM), the LightGBM model optimized using the PSO algorithm (PSO-LightGBM), the XGBoost model optimized using the IZOA proposed in this paper (IZOA-XGBoost), the default LightGBM model, Logistic Regression (LR), and a Multi-Layer Perceptron (MLP) with default hyperparameters. The optimal parameters of each optimization algorithm determined after several experiments are shown in Table 8. Both the LightGBM and XGBoost models used the multi-category log loss as their performance metric, suitable for evaluating models in multi-categorization tasks.

#### 4.4.1. Comparison and Analysis of Confusion Matrices

**Figure 9:**
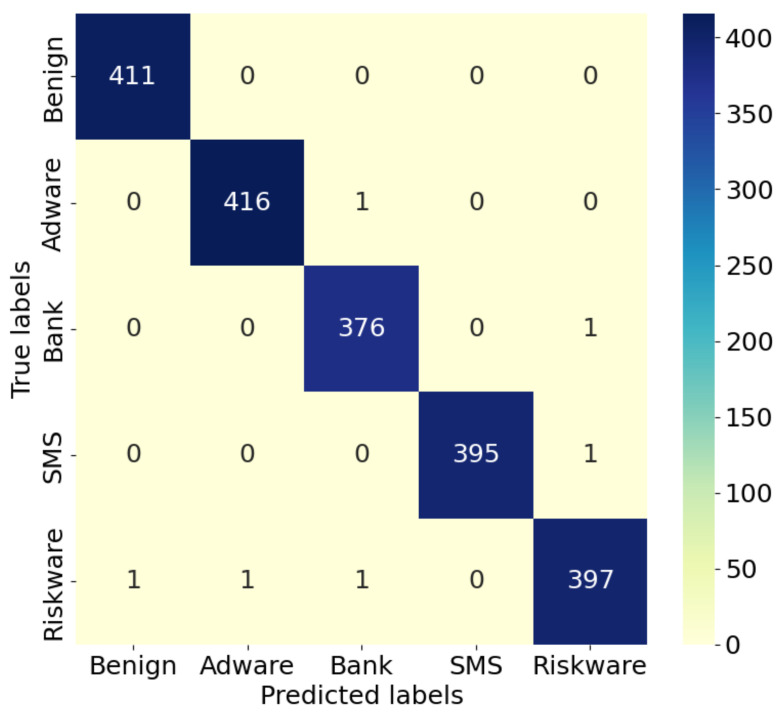
Confusion matrix of IZOA-LightGBM on CMD.

**Figure 10:**
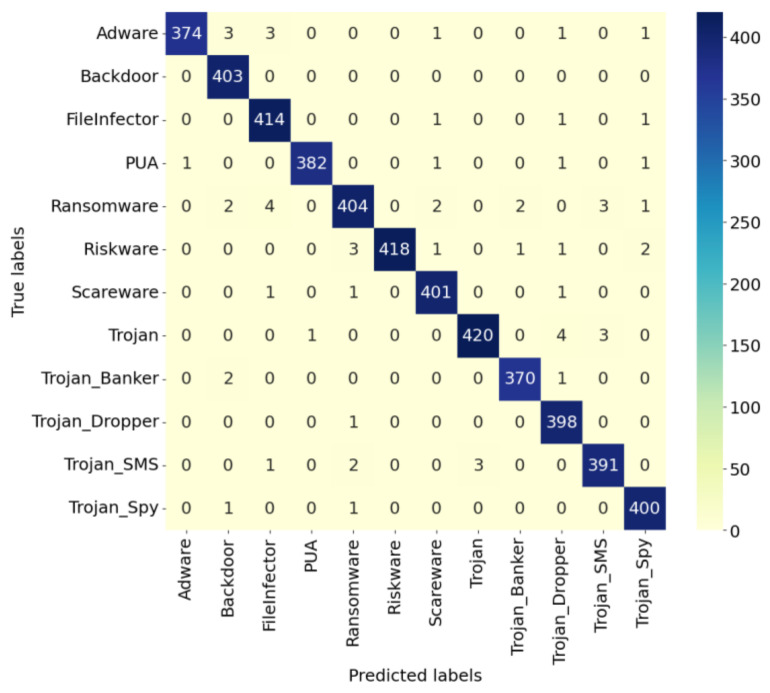
Confusion matrix of IZOA-LightGBM on CCA.

**Figure 11:**
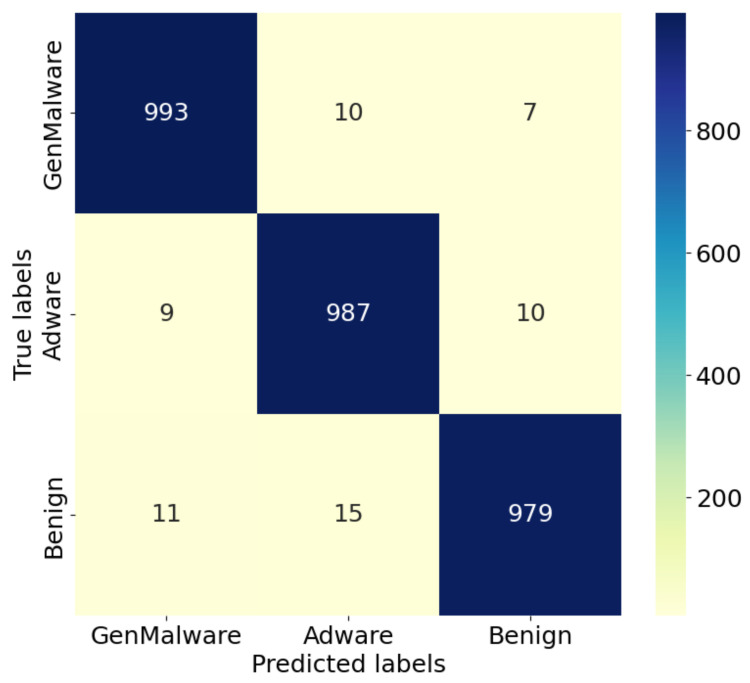
Confusion matrix of IZOA-LightGBM on AAGM.

The confusion matrix, as a commonly used performance evaluation method, can intuitively display model detection effects. It is shown for the IZOA-LightGBM model in Figure 9, Figure 10 and Figure 11.

As can be seen in Figure 9, Figure 10 and Figure 11, the majority of the IZOA-LightGBM model samples in this study were focused along the principal diagonal of the confusion matrix. Points on the primary diagonal of the matrix indicate correct classifications, whereas off-diagonal points signify misclassifications. Thus, the high percentage of main diagonal samples is intuitive evidence of the model’s superior detection performance. On the CMD dataset, the IZOA-LightGBM model incorrectly recognized only 5 out of 2000 samples in the test set. This outcome indicates that the model has a strong ability to distinguish between software samples when they are tested.

#### 4.4.2. Accuracy Comparison

Table 9 presents a comparative analysis of the overall accuracy of the eight models on the CMD, CCA, and AAGM datasets, while Table 10 provides a comparison of the accuracy for each individual class.

Table 9 shows that the IZOA-LightGBM model achieves higher overall accuracy on the CMD, CCA, and AAGM datasets, with 99.75%, 98.86%, and 97.95%, respectively. Table 10 indicates that the model also achieves higher accuracy for most categories. This suggests that the IZOA effectively enhances the classification capabilities of the LightGBM model. The accuracy of the LightGBM model combined with other optimization algorithms (ZOA, SA, PSO) is also higher than that of the unoptimized model. This indicates that these algorithms positively influence the model’s performance. However, the LR and MLP models do not perform as well as the decision tree-based detection models on any of the datasets, with particularly lower accuracies on the CMD dataset.

#### 4.4.3. Precision Comparison

Table 11 compares the precision of the aforementioned eight models on the CMD, CCA, and AAGM datasets.

As shown in Table 11, the IZOA-LightGBM model demonstrates superior precision across the CMD, CCA, and AAGM datasets compared to the other models. The ZOA-LightGBM, SA-LightGBM, and PSO-LightGBM models also performed similarly, but lower than the IZOA-LightGBM model in some categories. The IZOA-XGBoost model also performs better overall, but still slightly below the previously mentioned models. The LR and MLP models are less precise than the other models in most categories, probably because of the lack of hyperparameter optimization.

#### 4.4.4. Recall Rate Comparison

Table 12 compares the recall rate of the aforementioned eight models on the CMD, CCA, and AAGM datasets.

As shown in Table 12, the recall rate exhibits a similar trend to that of the precision metric. On the CMD dataset, the IZOA-LightGBM model performs better, with a recall rate at or near 100% for the Benign and Adware classes. Similarly, on the CCA dataset, the FileInfector and Backdoor categories have 100% recall rate. In contrast, unoptimized models like the default LightGBM, LR, and MLP models display inconsistent performance, performing well in some categories but not in others. In addition, for the FileInfector category, most of the models are correctly recognized. On the AAGM dataset, for the Benign category, the recall of the eight models was generally lower than the other two categories. This phenomenon may indicate that the models have some challenges in recognizing the Benign category.

#### 4.4.5. F1-Score Comparison

Table 13 shows the F1-score comparison of the aforementioned eight models on the CMD, CCA, and AAGM datasets.

In contrast to the previous precision and recall rate analyses, the F1-score, as a reconciled average of the two, provides a more comprehensive assessment of model performance. A high F1-score means that the model has a better balance between precision and recall rate. As shown in Table 13, the IZOA-LightGBM model performs better on the CMD and AAGM datasets and in most categories of the CCA dataset. Differences in the F1-scores across datasets can be observed, with the IZOA-LightGBM model showing superior overall performance on the CMD dataset compared to the CCA dataset. This disparity may be attributed to differences in sample distribution, feature representation, etc., which affect the model’s generalization ability. Moreover, experimental results indicate that an increase in the complexity of the sample categories may lead to increased detection difficulty.

#### 4.4.6. Loss Function Convergence Analysis

**Figure 12:**
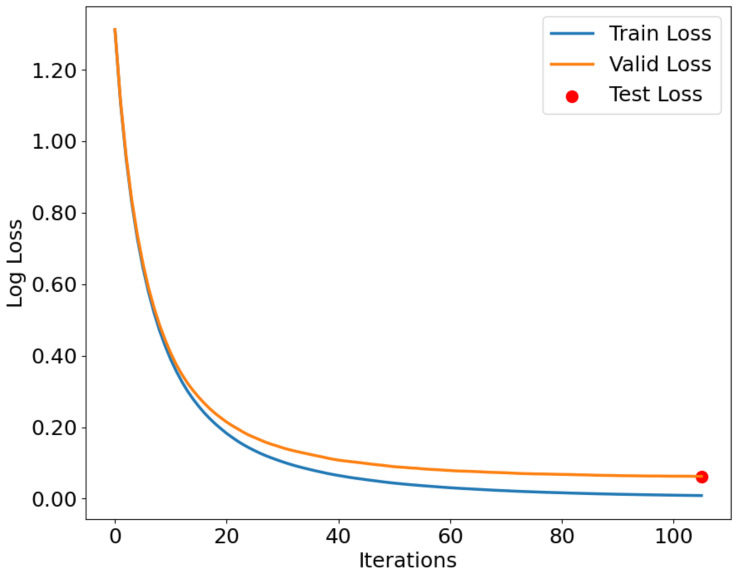
Loss function curve for CMD dataset.

**Figure 13:**
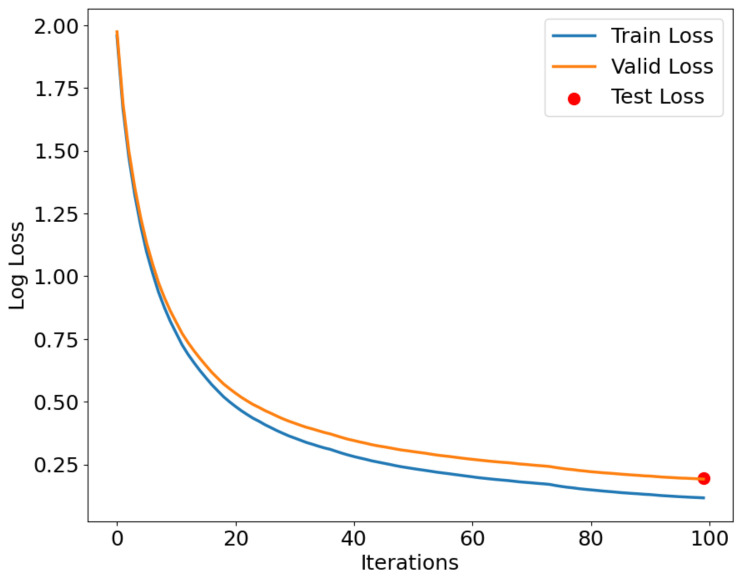
Loss function curve for CCA dataset.

**Figure 14:**
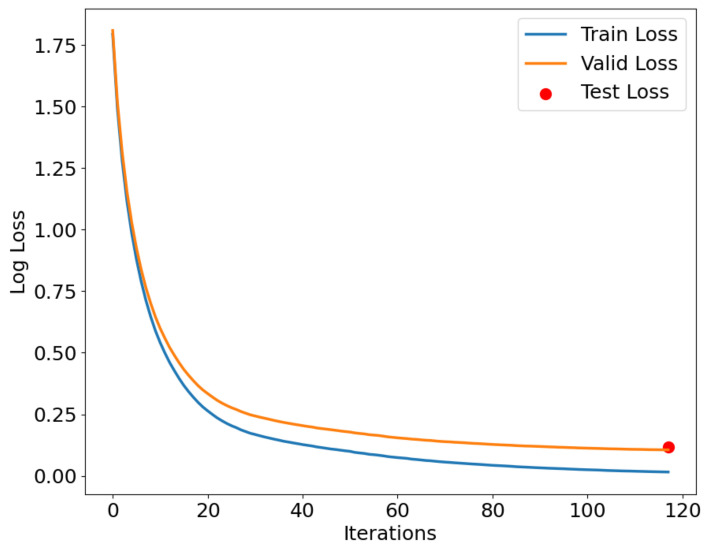
Loss function curve for AAGM dataset.

As shown in Figure 12, Figure 13 and Figure 14, the IZOA-LightGBM model’s loss function variation with the number of iterations for the training, validation, and testing processes on the CMD, CCA, and AAGM datasets is illustrated. The blue line indicates training loss, the orange line indicates validation loss, and the red dots indicate testing loss.

Figure 12, Figure 13 and Figure 14 show that both training and validation losses decrease as the iteration count increases, and then stabilize. The training loss is consistently lower than the validation loss, indicating that the model performs better on the training set than on the validation set. This phenomenon is expected because the model tries to fit the training data as much as possible during the training process. In addition, the gap between the validation loss and the training loss stays within a small range throughout the training process, indicating that the model does not undergo significant overfitting during the training process. The figure indicates that the test loss is approximately the final value of the validation loss, indicating that the model performs similarly on both sets.

#### 4.4.7. Comparison of Different Improvement Strategies 

This study verified the necessity of improving the IZOA-LightGBM model. The LightGBM model was optimized using the EOBL-improved ZOA (EZOA), the FDS-improved ZOA (FZOA), and the unimproved ZOA, respectively. The evaluation results are shown in Table 14.

Table 14 indicates that all the different improvement strategies improve the model detection capabilities. Compared with other improvement strategies, the IZOA-LightGBM model incorporates the improvements of both strategies and has better performance.

#### 4.4.8. Time Complexity and Model Size Comparison

The efficiency and deployment feasibility of the model were assessed by comparing the time complexity and model size, respectively. Table 15 presents the evaluation outcomes.

As shown in Table 15, the IZOA-LightGBM model has a higher time complexity, evident in its longer training duration. However, since the training was conducted offline and the resulting model can be reused, the training time is less critical. In addition, the shorter detection time and smaller model size of the IZOA-LightGBM model means that it may be suitable for deployment in devices with limited computing resources for the real-time detection of Android malware.

The LightGBM model optimized with a meta-heuristic algorithm shows improved detection performance on three datasets compared to the default hyperparameter configuration, but with significant increases in training time and model size. The increase in training time may be due to the introduction of the metaheuristic algorithm, and the increase in model size may be due to the larger and deeper trees generated by the LightGBM model. The SA-LightGBM model exhibited the longest training time on both datasets, probably due to the longer optimization time required for the SA. The IZOA-XGBoost model performs consistently on both datasets, but takes longer to train and detect, and it exhibits larger model sizes of 28.43 MB (CMD) and 37.69 MB (CCA), respectively. In addition, the models on the CCA dataset generally exhibit longer training times, which may be due to the fact that the CCA dataset has more sample categories and numbers than the CMD dataset.

#### 4.4.9. Comparison of This Work with Others

This paper reviews some additional relevant studies from the literature review. Most of the studies used ML or DL methods. Mahdavifar et al. [17], the proposers of the CMD dataset, proposed a Pseudo-Labeled Deep Neural Network (PLDNN) detection method and conducted experiments on the CMD dataset. Mohamed et al. [45] analyzed the CMD dataset using four different ML models. Musikawan et al. [40] proposed an effective improved DNN. Ullah et al. [46] proposed an integrated model. Rahali et al. [37] proposed an image-based DNN approach. Jundi et al. [47] proposed a detection model using a combination of XGBoost and Grammar Evaluation (GE). Xie et al. [48] proposed a DL-based model for multiple-label detection. Tang et al. [49] proposed an Android malware classification method based on Hybrid Bytecode Images (HBIs) and a DNN combined with an Attention Mechanism (DNN-AM). Al-Andoli et al. [50] proposed a parallel DL framework called PDL-FEMC. Li et al. [51] proposed SynDroid, a model for detecting Android malware. Alani et al. [52] proposed a malware detection framework called AdStop. Ullah et al. [53] proposed a semantics-driven Federated Learning (FL) approach for Android malware detection. The papers were categorized by dataset and sorted by time of proposal. Table 16 displays the results.

In Table 16, the studies by Musikawan et al. [40], Batouche et al. [56], and Xie et al [48]. are shown to be less accurate due to the inclusion of the two categories that the authors of the CCA dataset recommended for removal. Table 16 illustrates that the IZOA-Light model achieves higher accuracy than the models of Mahdavifar et al. [40] and Padmavathi et al. [55] on the CMD dataset, and of Rahali et al. [37] on the CCA dataset, under conditions where dynamic analysis was utilized and identical categories are detected. A similar trend was maintained on the AAGM dataset.

### 4.5. Summary

This chapter aimed to evaluate the availability of the IZOA-LightGBM model proposed in this paper in Android malware detection. The CMD, CCA, and AAGM datasets, which are three public datasets, were used to evaluate the detection performance of the IZOA-LightGBM model. First, the CMD and CCA datasets were preprocessed separately, including dataset balancing, feature selection, and feature dimensionality reduction, to improve the accuracy and efficiency during model training and detection. After that, both the IZOA-LightGBM model and the comparative models were evaluated in terms of accuracy, precision, recall rate, F1-score, training time, detection time, and model size. The evaluation results on the CMD, CCA, and AAGM datasets show that the IZOA-LightGBM model exhibits better detection performance compared to LightGBM models combined with other optimization algorithms such as the ZOA, SA, and PSO. In addition, the short detection time and moderate model size of the IZOA-LightGBM model may be suitable for deployment on real-time Android malware detection devices with limited computational resources.

## 5. Conclusions

Aiming to enhance the accuracy of Android malware detection via dynamic analysis, this paper proposes a novel model named IZOA-LightGBM. This model further improves the convergence speed and search capability of the ZOA by introducing EOBL and the FDS. Through optimizing the hyperparameters of the LightGBM model using the IZOA, the accuracy of the LightGBM model in detecting Android malware was further improved.

The validity of the proposed model was validated by extensive evaluation on three datasets. The evaluation results show that the IZOA-LightGBM model achieved an overall accuracy of 99.75% on the CMD dataset, 98.86% on the CCA dataset, and 97.95% on the AAGM dataset. It outperforms other comparative models, demonstrating the potential of the model for application in Android malware detection.

However, the model complexity needs to be further reduced, and enhancements can still be made to improve detection accuracy. Therefore, upcoming research will investigate additional efficient and compact detection models to enhance accuracy further. This research is preliminary, based on public datasets, and has not been tested in a live setting. Future work can deeply analyze the application of the model in real Android malware detection scenarios, as well as its performance with other types of malware and in different environments, such as malware in a Windows environment. Furthermore, future research will explore additional data sources and a broader range of features to enhance the model’s robustness. The influence of dataset bias on the model performance will also be assessed in future work. In addition, the overfitting of the model needs to be further considered in future work.

## Figures and Tables

**Table 1:** Hyperparameters for LightGBM model optimization.

Hyperparameters	Meaning
learning_rate	Learning rate
min_child_samples	Minimum number of samples required for leaf nodes
max_depth	Maximum depth
num_leaves	Number of leaf nodes in each tree
max_bin	Maximum possible number of eigenvalue bins
min_data_in_leaf	Minimum number of samples for leaf nodes
feature_fraction	Proportion of feature subsets used to train the model
bagging_fraction	Control the sampling ratio of the training data
bagging_freq	Frequency of bagging
reg_alpha	L1 regular term coefficient
reg_lambda	L2 regular term coefficient

**Table 2:** CMD dataset.

Name	Count
Adware	1253
Banking malware	2100
SMS malware	3904
Riskware	2546
Benign software	1795
Total	11,598

**Table 3:** CCA dataset.

Name	Count
Adware	5142
Backdoor	546
FileInfector	119
PUA	625
Ransomwore	1550
Riskware	6792
Scareware	424
Trojan	4025
Trojan_Banker	123
Trojan_Dropper	733
Trojan_SMS	911
Trojan_Spy	1039
Total	22,029

**Table 4:** AAGM dataset.

Name	Count
Benign	471,597
Adware	155,613
GenMalware	4745
Total	631,955

**Table 5:** Comparison of different oversampling techniques.

Datasets	Techniques	Accuracy (%)
CMD	ROA	**98.4**
	ADASYN	97.8
	SMOTE	97.3
	Unbalanced	93.8
CCA	ROA	**96.33**
	ADASYN	95.63
	SMOTE	95.21
	Unbalanced	85.51
AAGM	ROA	90.36
	ADASYN	**91.45**
	SMOTE	90.09
	Unbalanced	88.53

Bold indicates the highest accuracy.

**Table 6:** Feature selection results.

Datasets	Original Feature Count	Remaining Feature Count	Accuracy before Feature Selection (%)	Accuracy after Feature Selection (%)
CMD	471	230	98.4	**98.5**
CCA	142	115	96.33	**96.46**
AAGM	80	71	91.45	**91.77**

Bold indicates the highest accuracy.

**Table 7:** Comparison of dataset before and after dimensionality reduction.

Datasets		Feature Count	Accuracy (%)	Training Time (s)
CMD	Before Dimensionality Reduction	230	98.5	4.3
	After Dimensionality Reduction	29	97.8	1.0
CCA	Before Dimensionality Reduction	115	96.46	11.7
	After Dimensionality Reduction	108	95.94	10.5
AAGM	Before Dimensionality Reduction	80	91.77	5.6
	After Dimensionality Reduction	46	90.57	4.1

**Table 8:** Parameters of optimization algorithms.

Algorithms	Parameters	Value	Meaning
IZOA	pop	30	Population number
	maxiter	100	Maximum number of iterations
	dim	11	Problem dimension
	θ	2	Controls the amplitude of disturbance
	$\beta_{0}$	2	Initial attraction coefficient
	δ	1	Rate of attraction decay adjustment
ZOA	pop	30	Population number
	maxiter	100	Maximum number of iterations
	dim	11	Problem dimension
SA	maxiter	100	Maximum number of iterations
	dim	11	Problem dimension
	tolfun	1 × 10^−9^	Tolerance for change
PSO	ps	30	Number of particles
	maxiter	100	Maximum number of iterations
	dim	11	Problem dimension
	w	9	Inertia factor
	c1	2	Acceleration constant
	c2	2	Acceleration constant
	v_min	−5	Lower bound of velocity
	v_max	5	Lower bound of velocity

**Table 9:** Comparison of overall accuracy.

Model	Accuracy (%)		
	CMD	CCA	AAGM
IZOA-LightGBM	**99.75**	**98.86**	**97.95**
ZOA-LightGBM	98.76	97.76	96.44
SA-LightGBM	98.85	97.93	96.41
PSO-LightGBM	98.94	97.54	97.04
IZOA-XGBoost	98.40	97.47	96.97
LightGBM	97.8	95.94	90.57
LR	46.57	73.33	60.97
MLP	55.55	91.84	79.33

Bold indicates the highest accuracy.

**Table 10:** Comparison of each category’s accuracy.

Datasets	Category	Model							
		IZOA-LightGBM	ZOA-LightGBM	SA-LightGBM	PSO-LightGBM	IZOA-XGBoost	LightGBM	LR	MLP
CMD	Benign	**100**	99.27	99.51	99.27	99.51	96.35	59.37	69.59
	Adware	**99.76**	98.32	98.56	99	98.32	98.08	20.62	67.63
	Bank Malware	**99.73**	98.94	99.2	99.2	98.93	97.34	18.04	23.87
	SMS Malware	**99.75**	99.24	98.98	99.24	97.47	97.72	91.67	34.6
	Riskware	**99.5**	97.99	97.99	97.99	97.74	93.98	42.36	79.2
CCA	Adware	**97.65**	95.04	96.6	96.6	93.47	88..25	53.2	73.15
	Backdoor	**100**	99.75	99.75	**100**	99.75	99.75	70.8	99.74
	FileInfector	**100**	**100**	99.76	**100**	99.76	99.76	96.21	**100**
	PUA	**99.74**	**99.74**	99.48	99.48	99.73	99.73	80.61	99.05
	Ransomware	**96.65**	94.26	95.7	96.53	94.26	94.26	76.34	80.67
	Riskware	**98.12**	94.6	94.6	93.3	93.9	86.85	61.37	79.46
	Scareware	99.26	**100**	99.5	99	**100**	99.5	84.24	99.26
	Trojan	**98.13**	92.29	92.28	92.29	91.12	85	58.05	82.06
	Trojan_Banker	99.2	**99.73**	**99.73**	99.19	**99.73**	99.19	88.68	98.54
	Trojan_Dropper	**99.75**	99.74	99.74	99.74	99.72	99.19	58.37	98.14
	Trojan_SMS	**98.49**	99.24	98.74	98.48	99.23	99.24	72.66	94.27
	Trojan_Spy	**99.5**	99.49	99.49	98.5	99	98.5	80.09	95.65
AAGM	GenMalware	**98.31**	97.68	97.29	98.06	98.26	91.96	66.37	82.12
	Adware	**98.11**	96.62	96.51	97.21	97.01	93.03	63.98	82.19
	Benign	**97.41**	94.98	95.38	95.78	95.98	86.65	50.1	70.68

Bold indicates the highest accuracy.

**Table 11:** Precision comparison.

Datasets	Category	Model							
		IZOA-LightGBM	ZOA-LightGBM	SA-LightGBM	PSO-LightGBM	IZOA-XGBoost	LightGBM	LR	MLP
CMD	Benign	**99.76**	98.79	99.03	98.79	99.03	96.19	77.46	74.67
	Adware	**99.76**	98.8	98.8	98.8	98.09	97.56	53.75	65.89
	Bank Malware	**99.73**	98.42	98.68	98.94	97.39	98.33	72.34	79.65
	SMS Malware	**100**	98.5	98.49	98.99	98.47	99.74	31.62	82.53
	Riskware	**99.5**	99.24	99.24	99.24	98.98	96.9	59.72	34.73
CCA	Adware	**99.73**	97.59	97.63	97.63	97.55	92.1	64.86	86.59
	Backdoor	**98.05**	96.87	96.88	96.18	96.87	97.11	71.92	91.25
	FileInfector	97.87	99.28	99.04	97.87	99.28	99.04	81.41	**99.75**
	PUA	**99.74**	98.71	98.96	97.94	97.2	95.26	73.18	95.01
	Ransomware	**98.06**	97.04	96.62	96.53	97.04	97.04	65.93	94.35
	Riskware	**100**	99.02	99.02	99.02	98.52	92.04	74.04	86.9
	Scareware	**99.01**	98.78	98.53	98.52	98.78	98.78	86.15	97.58
	Trojan	**99.29**	97.29	97.29	97.29	96.53	91.46	71.66	83.83
	Trojan_Banker	99.2	**99.73**	**99.73**	98.93	99.47	99.47	74.07	99.22
	Trojan_Dropper	**98.03**	97.55	97.31	96.84	97.55	97.55	72.75	87.01
	Trojan_SMS	**98.49**	94.03	95.38	94.9	94.03	94.03	61.86	88.29
	Trojan_Spy	**99.01**	97.56	**99.01**	99	97.09	97.09	80.83	92.07
AAGM	GenMalware	**98.03**	96.01	96	96.48	96.4	91.07	70.75	82.12
	Adware	**97.53**	96.33	96.33	97.12	97.31	89.3	53.18	73.94
	Benign	**98.29**	97.03	96.94	97.55	97.25	91.42	60.85	83.01

Bold indicates the highest precision.

**Table 12:** Recall rate comparison.

Datasets	Category	Model							
		IZOA-LightGBM	ZOA-LightGBM	SA-LightGBM	PSO-LightGBM	IZOA-XGBoost	LightGBM	LR	MLP
CMD	Benign	**100**	99.27	99.51	99.27	99.51	96.75	59.37	69.59
	Adware	**99.76**	98.32	98.56	99.04	98.32	98.08	20.62	67.63
	Bank Malware	**99.73**	98.94	99.2	99.2	98.94	97.65	18.04	23.87
	SMS Malware	**99.75**	99.24	98.99	99.24	97.47	97.73	91.67	34.6
	Riskware	**99.5**	97.99	97.99	97.99	97.74	95.98	42.36	79.2
CCA	Adware	**97.65**	95.04	96.61	96.61	93.47	88.25	53.2	73.15
	Backdoor	**100**	99.75	**100**	**100**	99.75	**100**	70.8	99.74
	FileInfector	**100**	**100**	**100**	**100**	**100**	**100**	96.21	**100**
	PUA	**99.75**	99.74	99.48	99.48	99.74	99.74	80.61	99.05
	Ransomware	**96.65**	94.26	95.69	93.3	94.26	94.26	76.34	80.66
	Riskware	**98.12**	94.6	94.6	94.6	93.9	86.85	61.37	79.46
	Scareware	99.26	**100**	99.5	99.01	**100**	**100**	84.24	99.26
	Trojan	**98.13**	92.29	92.29	92.29	91.12	85.05	58.05	82.06
	Trojan_Banker	99.2	99.73	99.73	99.2	99.73	**100**	88.68	**100**
	Trojan_Dropper	**99.77**	99.75	99.75	99.75	99.75	99.75	58.37	98.14
	Trojan_SMS	98.49	99.24	98.74	98.49	**99.25**	99.24	72.66	94.27
	Trojan_Spy	**99.6**	99.5	99.5	98.51	99.5	99.5	80.09	95.65
AAGM	GenMalware	**98.32**	97.68	97.29	98.07	97.88	91.96	66.38	82.13
	Adware	**98.11**	96.62	96.52	97.21	97.01	93.03	64.95	83.43
	Benign	**97.41**	94.98	95.38	95.78	95.98	86.65	51.18	72.2

Bold indicates the highest recall rate.

**Table 13:** F1-score comparison.

Datasets	Category	Model							
		IZOA-LightGBM	ZOA-LightGBM	SA-LightGBM	PSO-LightGBM	IZOA-XGBoost	LightGBM	LR	MLP
CMD	Benign	**99.88**	99.03	99.27	99.03	99.27	96.77	67.22	72.04
	Adware	**99.76**	98.56	98.68	98.92	98.2	97.8	29.81	66.75
	Bank Malware	**99.73**	98.68	98.94	99.07	98.16	97.83	28.87	36.73
	SMS Malware	**99.87**	98.87	98.74	99.12	97.97	99.72	47.02	48.75
	Riskware	**99.5**	98.61	98.61	98.61	98.36	95.62	49.56	48.28
CCA	Adware	**98.68**	96.3	97.11	97.11	95.47	90.13	58.46	73.15
	Backdoor	**99.72**	98.29	98.41	98.05	98.29	98.53	71.35	99.02
	FileInfector	98.92	99.64	99.52	98.92	99.64	99.52	88.19	**100**
	PUA	**99.74**	99.22	99.22	98.7	98.45	97.45	76.72	99.05
	Ransomware	**97.35**	95.63	96.15	94.89	95.63	95.63	70.75	80.66
	Riskware	**99.05**	96.76	96.76	96.76	96.15	89.37	67.11	79.46
	Scareware	99.13	**99.38**	99.01	98.77	**99.38**	**99.38**	85.18	99.26
	Trojan	**98.71**	94.72	94.72	94.72	93.75	88.14	64.14	82.06
	Trojan_Banker	99.2	99.73	99.73	99.06	99.6	99.73	80.72	**100**
	Trojan_Dropper	**98.88**	98.64	98.51	98.27	98.64	98.64	64.77	98.14
	Trojan_SMS	**98.49**	96.57	97.03	96.66	96.57	96.57	66.83	94.27
	Trojan_Spy	**99.29**	98.52	99.26	98.75	98.28	98.28	80.46	95.65
AAGM	GenMalware	**98.17**	96.84	96.64	97.27	97.13	91.51	68.49	82.12
	Adware	**97.82**	96.47	96.42	97.17	97.16	91.13	58.48	78.4
	Benign	**97.85**	95.99	96.15	96.66	96.61	88.97	55.6	77.23

Bold indicates the highest F1-score.

**Table 14:** Comparison of different improvement strategies.

Model	Accuracy (%)		
	CMD	CCA	AAGM
IZOA-LightGBM	**99.75**	**98.86**	**97.95**
EZOA-LightGBM	99.02	97.98	97.66
FZOA-LightGBM	98.97	98.15	97.52
ZOA-LightGBM	98.76	97.76	96.44

Bold indicates the highest accuracy.

**Table 15:** Time complexity and model size comparison.

Datasets	Model	Training Time (s)	Detection Time (ms)	Model Size (MB)
CMD	IZOA-LightGBM	26,426.7	8.53	16.37
	ZOA-LightGBM	18,503.83	7.25	12.69
	SA-LightGBM	43,180.52	7.13	15.21
	PSO-LightGBM	31,810.4	6.55	18.99
	IZOA-XGBoost	33,885.88	13.56	28.43
	LightGBM	3.2	**6.89**	1.71
	LR	**2.67**	7.25	**0.26**
	MLP	6.42	15.77	9.03
CCA	IZOA-LightGBM	30,132.51	5.98	22.23
	ZOA-LightGBM	25,536.28	7.41	21.58
	SA-LightGBM	49,850.6	6.22	23.66
	PSO-LightGBM	40,734.69	7.48	25.87
	IZOA-XGBoost	30,854.32	10.56	37.69
	LightGBM	10.5	5.45	4.16
	LR	**3.62**	**3**	**0.37**
	MLP	52.52	6.99	11.53
AAGM	IZOA-LightGBM	28,265.45	4.56	20.56
	ZOA-LightGBM	26,894.79	3.55	21.05
	SA-LightGBM	50,897.62	6.59	22.14
	PSO-LightGBM	42,569.17	4.88	21.89
	IZOA-XGBoost	33,458.53	16.59	38.68
	LightGBM	4.53	3.05	4.27
	LR	**1.56**	**2**	**0.42**
	MLP	22.66	4.54	12.88

Bold indicates the shortest time or smallest model size.

**Table 16:** Comparison with other works.

Dataset	Reference	Year	Analysis	No. of Classes	Method	Accuracy (%)
CMD	Mahdavifar et al. [17]	2020	Dynamic	5	PLDNN	97.84
	Mohamed et al. [45]	2021	Static	2	NB, SVM, KNN, DT	86
	Ahmed et al. [54]	2022	Hybrid	5	RF, MLP	97.5
	Musikawan et al. [40]	2022	Static	2	DNN	98.18
			Dynamic	2	DNN	93.5
	Ullah et al. [46]	2022	Dynamic	4	NB, SVM, DT, LR, RF	99.11
	Padmavathi et al. [55]	2022	Dynamic	5	K-means, PCA	88
	Jundi et al. [47]	2023	Hybrid	5	XGBoost, GE	98
	Tang et al. [49]	2024	Hybrid	2	HBI, DNN-AM	98.67
	Proposed	2024	Dynamic	5	IZOA-LightGBM	**99.75**
CCA	Rahali et al. [37]	2020	Dynamic	12	Semi-Supervised Deep Learning	93.36
	Batouche et al. [56]	2021	Static	14	RF	89
	Musikawan et al. [40]	2022	Static	2	DNN	97.72
			Dynamic	14	DNN	78.82
	Al-Andoli et al. [50]	2022	Static	12	PDL-FEMC	97.6
	Xie et al. [48]	2023	Static	15	MLD-Model	83.17
	Islam et al. [57]	2023	Dynamic	12	Ensemble ML	95
	Li et al. [51]	2024	Static	12	SynDroid-RF	94.31
	Huang et al. [39]	2024	Hybrid	15	RF	88.2
	Proposed	2024	Dynamic	12	IZOA-LightGBM	**98.86**
AAGM	Bovenzi et al. [58]	2022	Dynamic	3	RF	97
	Alani et al. [52]	2022	Dynamic	2	AdStop	97.08
	Ullah et al. [53]	2024	Dynamic	3	FL	93.85
	Proposed	2024	Dynamic	3	IZOA-LightGBM	**97.79**

Bold indicates the highest accuracy.

## Data Availability

The CICMalDroid-2020 dataset is available at https://www.unb.ca/cic/datasets/maldroid-2020.html (accessed on 7 May 2024). The CCCS-CIC-AndMal-2020 dataset is available at https://www.unb.ca/cic/datasets/andmal2020.html (accessed on 7 May 2024). The methodology and code of this study, if required, should be obtained by contacting the corresponding author.
